# Effects of DL-Homocysteine Thiolactone on Cardiac Contractility, Coronary Flow, and Oxidative Stress Markers in the Isolated Rat Heart: The Role of Different Gasotransmitters

**DOI:** 10.1155/2013/318471

**Published:** 2013-11-24

**Authors:** Vladimir Zivkovic, Vladimir Jakovljevic, Olga Pechanova, Ivan Srejovic, Jovana Joksimovic, Dragica Selakovic, Nevena Barudzic, Dragan M. Djuric

**Affiliations:** ^1^Department of Physiology, Faculty of Medical Sciences, University of Kragujevac, Kragujevac, Serbia; ^2^Institute of Normal and Pathological Physiology and Centre of Excellence for Examination of Regulatory Role of Nitric Oxide in Civilization Diseases, Slovak Academy of Sciences, Bratislava, Slovakia; ^3^Institute of Medical Physiology “Richard Burian”, Faculty of Medicine University of Belgrade, Belgrade, Serbia

## Abstract

Considering the adverse effects of DL-homocysteine thiolactone hydrochloride (DL-Hcy TLHC) on vascular function and the possible role of oxidative stress in these mechanisms, the aim of this study was to assess the influence of DL-Hcy TLHC alone and in combination with specific inhibitors of important gasotransmitters, such as L-NAME, DL-PAG, and PPR IX, on cardiac contractility, coronary flow, and oxidative stress markers in an isolated rat heart. The hearts were retrogradely perfused according to the Langendorff technique at a 70 cm H_2_O and administered 10 **μ**M DL-Hcy TLHC alone or in combination with 30 **μ**M L-NAME, 10 **μ**M DL-PAG, or 10 **μ**M PPR IX. The following parameters were measured: *dp/dt* max, *dp/dt* min, SLVP, DLVP, MBP, HR, and CF. Oxidative stress markers were measured spectrophotometrically in coronary effluent through TBARS, NO_2_, O_2_^−^, and H_2_O_2_ concentrations. The administration of DL-Hcy TLHC alone decreased *dp/dt* max, SLVP, and CF but did not change any oxidative stress parameters. DL-Hcy TLHC with L-NAME decreased CF, O_2_^−^, H_2_O_2_, and TBARS. The administration of DL-Hcy TLHC with DL-PAG significantly increased *dp/dt* max but decreased DLVP, CF, and TBARS. Administration of DL-Hcy TLHC with PPR IX caused a decrease in *dp/dt *max, SLVP, HR, CF, and TBARS.

## 1. Introduction

Cardiovascular diseases (CVDs) are still the leading cause of morbidity and mortality among all racial and ethnic populations [1]. Most studies investigating this issue concluded that only a little more than half of the cases of CVDs can be linked with some of the classic risk factors [2]. Intensive research in this field in the last two decades has paid more attention to the sulphur-containing amino acid homocysteine (Hcy) as a risk factor for developing CVDs and labelled this molecule as the “cholesterol of the 21st century” [3]. Numerous epidemiological studies have shown a high association between hyperhomocysteinemia and increased risk for CVDs, thus promoting Hcy as a new and independent risk factor for these diseases [4, 5].

Several studies have shown that the L form of Hcy has the highest bioactive potential. However, the presence of a thiolactone group produces the highest toxicity of Hcy compounds, most likely through N-homocysteinylation [6] and inhibition of Na^+^, K^+^-ATPase [7]. The fate of Hcy thiolactone in cultures of human cells and its reactivity toward proteins and amino acids under physiological conditions were studied by Jakubowski [6]. The data suggested a mechanism by which Hcy, through its metabolic conversion to thiolactone, which in turn acylates proteins, can lead to cell damage resulting in pathology such as avascular disease. Hyperhomocysteinaemia represents a metabolic disorder caused by a deficiency of certain enzymes and/or vitamins that are involved in the homocysteine metabolic pathway. It causes accumulation of homocysteine in the blood [8, 9]. It has been reported that homocysteine evokes endothelial dysfunction and impairment of nitric oxide (NO) bioavailability in animal models [10] and cell culture studies [11]. One possible mechanism of homocysteine's effects is the generation of hydrogen peroxide (H_2_O_2_) [12] and the superoxide anion, which increases the oxidative degradation of NO [13]. The most widely known endothelium-derived relaxing factor, NO, is released from endothelial cells in response to shear stress or the stimulation of different receptors for a variety of neurohumoral mediators on the endothelial cell surface [14]. 

Nitric oxide synthases (NOSs) are a family of enzymes catalysing the production of nitric oxide (NO) from L-arginine. Nitric oxide (NO) impairs contractility [15], while an increased myocardial production of NO is proposed as a contributor to the progression of chronic cardiac failure [16]. Chronically failing hearts display an increased expression of nitric oxide synthase II (NOS II) [17], which leads to increased cardiac NO production [18]. Hemodynamic effects are accompanied by a significant decrease in nitrite outflow after *N* ^*ω*^-Nitro-L-arginine methyl ester (L-NAME) administration [19, 20]. Inactivation of nitric oxide (NO) by superoxide and other reactive oxygen species (ROS) seems to occur in conditions such as hypertension, hypercholesterolemia, diabetes, and cigarette smoking.

Hydrogen sulphide (H_2_S) is a signalling molecule that belongs to the gasotransmitter family. H_2_S is a potent vasodilator and has powerful anti-inflammatory, antioxidant and antiapoptotic effects [21–25], which are mediated by its ability to directly scavenge ROS and downregulate the ROS-producing enzymes. Three major endogenous sources of enzymatically produced H_2_S are cystathionine beta synthase (CBS), cystathionine gamma lyase (CSE), whose expression was shown in the cardiovascular system, and 3-mercaptopyruvate sulfurtransferase (MST). DL-Propargylglycine (DL-PAG) is an irreversible inhibitor of the H_2_S-synthesising enzyme cystathionine gamma lyase (CSE). Heme oxygenase (HO), a rate-limiting enzyme in heme metabolism, degrades heme into biliverdin/bilirubin, with the production of carbon monoxide (CO) and free iron (Fe). The products of heme metabolism produce various beneficial physiological effects, such as antioxidant effects, antiapoptotic effects, anti-inflammatory effects, vasodilation, cell cycle regulation, enhanced insulin sensitivity, adiponectin induction, and angiogenesis regulation [26, 27]. Heme oxygenases mainly include two isoenzymes: HO-1 and HO-2. HO-1 is an inducible isoenzyme whose expression and activity can be upregulated by inducers or downregulated by inhibitors, such as zinc protoporphyrin IX (ZnPPR IX). By using DL-PAG as an irreversible inhibitor of CSE and/or ZnPPR IX as an HO-1 inhibitor, we could indirectly examine the production and thus a potential role in cardiac function and coronary circulation of H_2_S and CO.

Considering the data regarding the adverse effects of DL-Hcy TLHC on vascular function and the role of oxidative stress in these mechanisms, the aim of this study was to evaluate its influence on cardiac contractility, coronary flow, and oxidative stress markers in the isolated rat heart.

## 2. Material and Methods

The hearts of male Wistar albino rats (*n* = 48,12 in each experimental group, BM 180–200 g) were excised and perfused according to the modified Langendorff technique at constant pressure conditions (Experimetria Ltd., Budapest, Hungary), as described previously [28]. Briefly, under ether anaesthesia, animals were premedicated with heparin as an anticoagulant and sacrificed by cervical dislocation (Schedule 1 of the Animals/Scientific Procedures, Act 1986, UK). After emergency thoracotomy and rapid cardiac arrest by superfusion with ice-cold isotonic saline, the hearts were rapidly excised; the aortas were cannulated and retrogradely perfused at the constant pressure (CPP) of 70 cm H_2_O. The composition of the nonrecirculating Krebs-Henseleit perfusate was as follows mM/L: NaCl 118, KCI 4.7, CaCl_2_·2H_2_O 2.5, MgSO_4_·7H_2_O 1.7, NaHCO_3_ 25, KH_2_PO_4_ 1.2, glucose 11, and pyruvate 2, equilibrated with 95% O_2_ plus 5% CO_2_ and warmed to 37°C (pH 7.4). Immediately after normal heart rhythm returned, the sensor (transducer BS4 73-0184, Experimetria Ltd., Budapest, Hungary) was inserted through the newly damaged left atrium and mitral valve into the left ventricle for continuous monitoring of cardiac function.

### 2.1. Physiological Assay and Experimental Protocol

To test coronary vascular reactivity, all hearts were challenged by short-term occlusions (5–30 s), followed by a bolus injection of 5 mM/L adenosine (60 *μ*L at a flow rate of 10 mL/min to elicit maximum coronary flow (CF)) during the stabilisation period. Hearts were discarded if the flow did not increase by 100% over the control value for both tests (approximately 25% of hearts). Coronary flow was measured using flowmetry. When the flow was considered stable (three measurements of the same values), coronary effluent samples were collected. Only groups of hearts in which the CPP/CF relationship was studied twice in the absence of drugs were included in the study. After perfusion in the absence of any medication (control conditions), hearts were perfused with:

1. 10 *μ*M DL-Hcy thiolactone-hydrochloride (DL-Hcy TLHC);
2. 10 *μ*M DL-Hcy TLHC + 30 *μ*M L-NAME (*N* ^*ω*^-Nitro-L-arginine methyl ester, an inhibitor of NOS);
3. 10 *μ*M DL-Hcy TLHC + 10 *μ*M DL-PAG (DL-Propargylglycine, an inhibitor of cystathionine gamma lyase-CSE);
4. 10 *μ*M DL-Hcy TLHC + 10 *μ*M ZnPPR IX (protoporphyrin IX zinc, an inhibitor of HO-1) and compared to the respective controls.

By placing the sensor in the left ventricle, the following parameters of myocardial function were continuously registered:

1. maximum rate of pressure development in the left ventricle (*dp/dt* max);
2. minimum rate of pressure development in the left ventricle (*dp/dt* min);
3. systolic left ventricular pressure (SLVP);
4. diastolic left ventricular pressure (DLVP);
5. mean blood pressure (MBP);
6. heart rate (HR).

Coronary flow (CF) was measured using the flowmetric method.

All research procedures were approved by the Ethical Committee for Animal Welfare, Faculty of Medical Sciences, University of Kragujevac, Serbia.

### 2.2. Biochemical Assays

Oxidative stress parameters (index of lipid peroxidation measured as thiobarbituric acid reactive substances (TBARS), the superoxide anion radical O_2_^−^, hydrogen peroxide H_2_O_2_, and nitrite NO_2_^−^) were determined in coronary venous effluent samples using the spectrophotometric method (Specord S-600 Analytik Jena).

#### 2.2.1. Index of Lipid Peroxidation (Thiobarbituric Acid Reactive Substances (TBARS))

The degree of lipid peroxidation in the coronary venous effluent was estimated by measuring thiobarbituric acid reactive substances (TBARS) using 1% thiobarbituric acid (TBA) in 0.05 NaOH incubated with the coronary effluent at 100°C for 15 min and read at 530 nm. Krebs-Henseleit solution was used as a blank probe [29].

#### 2.2.2. Nitrite Determination

Nitric oxide rapidly decomposes to form stable metabolite nitrite/nitrate products. The nitrite level (NO_2_) was measured as an index of NO production using the Griess reagent. A total of 0.5 mL of perfusate was precipitated with 200 *μ*L of 30% sulphosalicylic acid, vortexed for 30 min, and centrifuged at 3,000 ×g. Equal volumes of the supernatant and Griess reagent, containing 1% sulphanilamide in 5% phosphoric acid/0.1% naphthalene ethylenediamine-dihydrochloride, were added, incubated for 10 min in the dark, and read at 543 nm. The nitrite levels were calculated using sodium nitrite as the standard [30].

#### 2.2.3. Determination of Superoxide Anion Radical

The level of the superoxide anion radical (O_2_^−^) was measured by Nitro Blue Tetrazolium (NBT) reaction in TRIS-buffer with coronary venous effluent and read at 530 nm. Krebs-Henseleit solution was used as a blank probe [31].

#### 2.2.4. Determination of H_2_O_2_

The measurement of H_2_O_2_ is based on the oxidation of Phenol Red by H_2_O_2_ in a reaction catalysed by horseradish peroxidase (HRPO) [32]. A volume of 200 *μ*L of perfusate was precipitated with 800 *μ*L of fresh Phenol Red solution (PRS), along with 10 *μ*L of 1 : 20 HRPO (made ex tempore). An adequate volume of Krebs-Henseleit solution was used for a blank probe (instead of coronary venous effluent). The level of H_2_O_2_ was measured at 610 nm.

### 2.3. Drugs

All drugs were purchased from Sigma-Aldrich Chemie GmbH, Germany.

### 2.4. Statistical Analysis

Values are expressed as the mean ± SE. Statistical analysis was performed by the Wilcoxon test. *P* values less than 0.05 were considered significant.

## 3. Results

### 3.1. The Effects of DL-Hcy TLHC, DL-Hcy TLHC + L-NAME, DL-Hcy TLHC + DL-PAG, or DL-Hcy TLHC + PPR IX on Myocardial Function Parameters in the Isolated Rat Heart

The administration of DL-Hcy TLHC (10 *μ*M) induced a significant decrease in *dp/dt* max (*P* < 0.05), SLVP (*P* < 0.01), and CF (*P* < 0.05) compared with control conditions. Other measured myocardial function parameters remained unchanged (Table 1(a)). Perfusion with DL-Hcy TLHC (10 *μ*M) + L-NAME (30 *μ*M) significantly decreased only CF (*P* < 0.01) (Table 1(b)). The application of DL-Hcy (10 *μ*M) + DL-PAG (10 *μ*M) induced a significant increase in *dp/dt* max (*P* < 0.05), a significant decrease in DLVP (*P* < 0.01), and a significant decrease in CF (*P* < 0.05) compared with control conditions. In contrast, this compound did not significantly affect *dp/dt* min, SLVP, HR, or MBP (Table 1(c)). The administration of DL-Hcy TLHC (10 *μ*M) + PPR IX (10 *μ*M) induced a significant decrease in *dp/dt* max (*P* < 0.05), SLVP (*P* < 0.05), HR (*P* < 0.05), and CF (*P* < 0.05) compared with the control conditions; *dp/dt* min was the only significantly increased parameter in this group of experiments under the influence of DL-Hcy TLHC (10 *μ*M) + PPR IX (10 *μ*M) (*P* < 0.05).

### 3.2. The Effects of DL-Hcy TLHC, DL-Hcy TLHC + L-NAME, DL-Hcy TLHC + DL-PAG, or DL-Hcy TLHC + PPR IX on Oxidative Stress Markers in the Isolated Rat Heart

Perfusion with DL-Hcy TLHC (10 *μ*M) did not induce significant changes in any oxidative stress markers (O_2_^−^, H_2_O_2_, NO_2_^−^, and TBARS) compared with control conditions (Table 2(a)). Perfusion with DL-Hcy TLHC (10 *μ*M) + L-NAME (30 *μ*M) induced significant decreases in O_2_^−^ (*P* < 0.05), H_2_O_2_ (*P* < 0.05), and TBARS (*P* < 0.01) compared with control conditions (Table 2(b)).

After heart perfusion with DL-Hcy (10 *μ*M) + DL-PAG (10 *μ*M), there was no significant difference in the levels of oxidative stress markers, except in the TBARS values, which were significantly decreased (*P* < 0.05) compared with control conditions (Table 2(c)). After heart perfusion with DL-Hcy TLHC (10 *μ*M) + PPR IX (10 *μ*M), there was no significant difference in the levels of oxidative stress markers, except in the TBARS values which were significantly decreased (*P* < 0.05) compared with control conditions (Table 2(d)).

## 4. Discussion

The aim of the present study was to assess the influence of acute administration of DL-Hcy TLHC and DL-Hcy TLHC in combination with specific inhibitors of important gasotransmitters, such as L-NAME (an inhibitor of NO production via inhibition of NOS), DL-PAG (an inhibitor of H_2_S production via inhibition of CSE), and PPR IX (an inhibitor of CO production via inhibition of HO-1), on cardiac contractility, coronary flow, and oxidative stress markers in the isolated rat heart. This study is partly a result of our investigation on the effects of Hcy and Hcy-related compounds on the cardiovascular system with special focus on gasotransmitters.

In the first part of our research, we focused on the effects of investigated compounds on dynamic parameters of myocardial function (*dp/dt* max, *dp/dt* min, SLVP, DLVP, MBP, HR, and CF). Our data showed that the administration of 10 *μ*M DL-Hcy TLHC induced depression of cardiac contractility in the isolated rat heart, manifested as a decrease in *dp/dt* max. The administration of DL-Hcy TLHC also induced a decrease in SLVP.

The results of this study correlate with the results of Moshal et al. [33], who found that an excess of Hcy-induced fibrosis and endothelial myocyte disconnection led to a decrease in myocardial contractility in a mouse model. Cardiomyocytes express NMDA receptors whose activation increases oxidative stress and Ca^2+^ load in the mitochondria, leading to cell death [34]. Hcy induces contractile dysfunction by an unknown mechanism. Moshal et al. [35] demonstrated a correlation between cardiomyocyte NMDA receptors, mitochondrial matrix metalloproteinases (mMMPs), and hyperhomocysteinemia. They showed that Hcy caused contractile dysfunction by activating mMMP and NMDA-R1. Our results show a decrease in SLVP by acute administration of DL-Hcy TLHC, in accordance with the results of Zivkovic et al. [28], who found that left ventricular systolic function significantly decreased after acute administration of Hcy compounds in rats. However, it is still unclear how Hcy compounds cause left ventricular systolic dysfunction.

Furthermore, our results show that DL-Hcy TLHC decreased SLVP in the isolated rat heart, in accordance with the results of previous investigations of acute and chronic models of hyperhomocysteinemia [28, 36]. Other cardiodynamic parameters were not significantly different under the influence of DL-Hcy TLHC (Table 1(a)).

DL-Hcy TLHC causes 10-fold increases in plasma homocysteine levels [37], according to the definition of referential ranges proposed in human and experimental models. Consequently, it would be expected that administration of DL-Hcy TLHC is more toxic than Hcy alone. However, Hcy may induce oxidative damage of endothelial cells, promote vascular smooth muscle growth, and inhibit regeneration of endothelial cells [38].

The inhibition of endothelial cell growth may be the result of methylation inhibition by Hcy [39]. In addition, Hcy may affect blood clotting mechanisms, thereby enchasing a prothrombotic state [40]. However, in the most cited experiments that led to this hypothesis, nonphysiological concentrations of Hcy (1–10 mmol/L) showed similar effects with cysteine or 2-mercaptoethanol [40, 41]. Although effects of Hcy on growth and methylation were found at physiological Hcy concentrations, these effects could only be observed in the presence of high levels of adenosine [39]. In this study, DL-Hcy TLHC did not affect cardiodynamic variables such as *dp/dt* min, DLVP, MBP, and HR.

In our research, acute administration of DL-Hcy TLHC did not significantly affect HR, but there are studies suggesting that chronic application of Hcy increased HR in rats by decreasing the elastin/collagen ratio in the left ventricle [42, 43]. Additionally, acute administration of DL-Hcy does not affect HR [44]. The acute administration of Hcy-related compounds in this model does not achieve sufficient concentrations to increase LV collagen expression and cause endocardial precapillary and interstitial fibrosis [42].

In the present study, we showed that DL-Hcy TLHC alone and in combination with L-NAME, DL-PAG, or PPR IX caused vasoconstriction and a consequent decrease in CF. One possible mechanism by which these compounds contribute to vasoconstriction is impaired relaxation of the vascular muscle. As will be described below, NO_2_^−^ as an indicator of NO production did not change significantly after the administration of any compound that we investigated. Based on this result, it can be suggested that in this experimental model, vasoconstriction could be induced mainly by acute effects of DL-Hcy TLHC and seems to be independent of NO release or the release of H_2_S and CO, as discussed below. However, studies investigating aortas in rats showed that the HO-1/CO pathway can have a role in the regulation of vascular tone [45].

The administration of L-NAME (an inhibitor of NOS) in combination with DL-Hcy TLHC was used to examine a potential effect of NO on cardiodynamic parameters and its influence on oxidative stress. The application of DL-Hcy TLHC induced significant changes in *dp/dt* max, SLVP, and CF, while the administration of DL-Hcy TLHC + L-NAME only affected CF, which decreased significantly. Hcy-thiolactone is generated in an error-editing reaction of protein biosynthesis when Hcy is selected instead of methionine by methionyl-tRNA synthetase10. Hcy-thiolactone, known to be cytotoxic in experimental animals and cell cultures, is detrimental mostly because of its ability to form isopeptide bonds with protein lysine residues (N-homocysteinylation) [6, 39, 46], which impairs or alters the protein function [47–50]. N-homocysteinylation increases protein susceptibility to oxidative damage [49] via a thiyl radical mechanism [51], causes formation of toxic amyloid-like protofibrils [52], and induces an autoimmune response [53]. Additionally, N-homocysteinylation can play a role in atherothrombosis through the accumulation of prothrombotic N-Hcy-fibrinogen [50] in CBS-deficient patients [54] and the accumulation of IgG anti-N-Hcy-protein autoantibodies in cardiovascular disease [55] and stroke [56] patients.

Originally discovered ex vivo in cultured human fibroblasts and endothelial cells, Hcy-thiolactone [57–59] is known to occur in vivo in humans and mice and to increase in nutritional or genetic hyperhomocysteinemia. Human umbilical endothelial cells have been reported to possess Hcy-thiolactone hydrolysing activity [38]. With regard to this fact, our aim was to evaluate the effects of DL-Hcy TLHC in the presence of NOS blockade by L-NAME. It seems that L-NAME abolished the effects of DL-Hcy TLHC on all cardiodynamic parameters except CF (Table 1(b)), suggesting that NOS blockade expresses protective effects in the heart via the improvement of contractile function compared to the application of DL-Hcy TLHC alone (Table 1(a)). Additional lowering of CF by simultaneous application of DL-Hcy TLHC and L-NAME compared to DL-Hcy TLHC alone can be a consequence of NO blockade (caused by L-NAME) and vasoconstriction of coronary vessels. 

We investigated the influence of CO by blocking endogenous production of this gasotransmitter via HO using ZnPPR IX. Similarly, DL-Hcy TLHC alone and DL-Hcy TLHC + PPR IX induced decreases in *dp/dt* max, SLVP, and CF. The combination of these compounds also affected *dp/dt* min and HR. There was an increase in *dp/dt* min approaching to the zero level, which shows an impaired cardiac contractility. HR most likely decreased due to the prolongation of action potentials and the reduction of the sinus rhythm rate. Our results for HR, (Table 1(d)) but not cardiac contractility, are similar to those of Abramochkin et al. [60].

In the heart, H_2_S is produced in the myocardium fibroblasts and blood vessels from L-cysteine by the enzyme CSE, which exerts an important effect on physiological and pathophysiological processes. The role of H_2_S has been investigated by using two main approaches: inhibiting endogenous H_2_S and administrating exogenous H_2_S, mainly using NaHS as a donor. In this research, we used DL-PAG to inhibit endogenous production of H_2_S. H_2_S-induced vasodilation is hypothesised to occur in ATP-sensitive potassium channels (K_ATP_), thus increasing cGMP levels in tissues. Bucci et al. suggest that H_2_S relaxes blood vessels by modulating cGMP levels [61]. Our results show a significant decrease in DLVP values, which correlates with the results obtained by Huang et al. [62], while *dp/dt* max values were opposite to those in the mentioned study (Table 1(c)). The reasons for these differences in *dp/dt* max levels remain unknown.

In the second part of our investigation, we explored whether acute administration of DL-Hcy.

TLHC and its combination with L-NAME, DL-PAG, or PPR IX promoted oxidative stress in the isolated rat heart by measuring oxidative stress parameters (O_2_^−^, H_2_O_2_, NO_2_^−^, and TBARS). In our previous research [35], there were no statistically significant changes in redox status in the isolated rat heart treated with DL-Hcy TLHC, which correlates with the results of the present study (Table 2(a)).

Additional application of DL-Hcy TLHC and L-NAME was followed by a significant reduction of all oxidative stress parameters, except NO_2_^−^ (Table 2(b)). This result is in accordance with some of our previous data [63, 64] showing decreased production of ROS after NOS inhibition following acute administration. This result can be a direct consequence of decreased O_2_^−^ production due to NOS inhibition, which leads to the direct lowering of H_2_O_2_ content and decreased global oxidative damage, measured as TBARS. The limitation of this study lies in the low-dose acute administration. We assume that chronic administration and higher exposure doses could result in different dynamics of ROS (increased generation of ROS).

Recently, H_2_S has been shown to protect rat cortical neurons from oxidative insult by stimulating GSH synthesis [65], reducing production of peroxynitrite [23], and reducing H_2_O_2_ [66]. It is important to note that so far, the antioxidant properties of H_2_S have been shown in the central nervous system [24] as a consequence of CBS action. Furthermore, recent results from Módis et al. [67] suggest antioxidant properties of this molecule due to the action of 3-mercaptopyruvate sulfurtransferase (3-MST), a novel enzyme identified in H_2_S-generating systems. The antioxidant properties of H_2_S produced in mitochondria are specific [68]. This intracellular action reduces intracellular oxidative stress parameters, with special focus on reducing oxidation of cysteine residues contained in intracellular enzymes [69]. Moreover, this interaction is reciprocal; the increased ROS production inactivates H_2_S [70, 71]. Interestingly, there are very limited data on the interaction between redox balance and the activity of CSE, a dominant H_2_S-producing enzyme in the cardiovascular system. According to our data (Table 2(c)), this effect is only present on the level of global oxidative damage, presented as TBARS. Namely, the DL-Hcy TLHC-induced changes of some oxidative stress parameters were not significantly affected by the simultaneous application of DL-Hcy TLHC + DL-PAG (an inhibitor of CSE). This result, which can be discussed as a consequence of CSE inhibition, shows an insignificant role of H_2_S produced by this enzyme in redox balance in the isolated rat heart.

CO is a gasotransmitter with a similar capacity as NO to bind heme proteins, with many common downstream-signalling pathways and functions. However, the role of CO in the presence or absence of NO may be unpredictable and diverse, depending on the concentration and tissue type [72]. One recent study by Soni et al. [73] showed a reduced I/R injury in the isolated rat heart under the influence of an exogenous CO donor in the presence of L-NAME. This result suggests that CO-induced cardioprotection is completely independent of NO. Our experimental model is focused on endogenous production of CO and its role in heart function. In addition, in our research, gasotransmitters are investigated only as a part of the heart response to DL-Hcy TLHC application. Additional blockade of endogenous CO production by PPR IX does not significantly change the primary effects of DL-Hcy TLHC on oxidative stress parameters. Only H_2_O_2_ was reduced by the simultaneous application of DL-Hcy TLHC + PPR IX (Table 2(c)), which made us conclude that this pathway did not contribute significantly to redox balance under the primary influence of the most toxic Hcy compound, DL-Hcy TLHC.

## 5. Conclusions

In summary, our findings clearly suggest that acute administration of DL-Hcy TLHC induces significant reduction of CF and partly of heart contractility, which confirms its cardiodepressive effect. The simultaneous application of different inhibitors of important cardiovascular gasotransmitters with DL-Hcy TLHC shows that additional HO-1 inhibition induces more powerful effects than NOS or CSE inhibition. The inhibition of CO production significantly increases DL-Hcy TLHC-induced effects on cardiodynamic parameters, while NOS and CSE inhibition only affects CF. Our results also suggest that acute administration of DL-Hcy TLHC and different gasotransmitter inhibitors does not show prooxidant potential. Furthermore, we did not find a link between cardiac dysfunction and oxidative stress after acute administration of DL-Hcy TLHC. Similar to our recent study, the data suggest that negative effects of DL-Hcy TLHC on myocardium do not necessarily involve oxidative stress.

## Figures and Tables

**Table 1a:** (a)

	*dp*/*dt* max (mmHg/s)	*dp*/*dt* min (mmHg/s)	SLVP (mmHg)	DLVP (mmHg)	MBP (mmHg)	HR (bpm)	Flow (mL/min)
Control (*X* ± SE)	2590.8 ± 160.7	−836.3 ± 358.7	70.1 ± 3.8	7.1 ± 3.5	50.4 ± 0.3	258.2 ± 16.2	11.5 ± 0.6
DL-Hcy TLHC (*X* ± SE)	2222.8 ± 231.0*	−1292.8 ± 177.9	53.9 ± 4.8**	2.9 ± 0.8	50.3 ± 0.2	265.9 ± 15.3	10.7 ± 0.6*

The values are expressed as the mean ± SE. *Statistical significance (*P* < 0.05), **high statistical significance (*P* < 0.01).

**Table 1b:** (b)

	*dp*/*dt* max (mmHg/s)	*dp*/*dt* min (mmHg/s)	SLVP (mmHg)	DLVP (mmHg)	MBP (mmHg)	HR (bpm)	Flow (mL/min)
Control (*X* ± SE)	1856.33 ± 259.37	−1112.63 ± 161.45	67.38 ± 5.23	16.10 ± 4.12	51.25 ± 1.78	229.65 ± 7.82	11.10 ± 0.84
DL-Hcy TLHC + L-NAME (*X* ± SE)	1691.35 ± 392.89	−1104.23 ± 318.24	57.11 ± 8.21	14.50 ± 3.76	50.16 ± 0.69	225.56 ± 7.33	8.10 ± 1.06**

The values are expressed as the mean ± SE. **High statistical significance (*P* < 0.01).

**Table 1c:** (c)

	*dp*/*dt* max (mmHg/s)	*dp*/*dt* min (mmHg/s)	SLVP (mmHg)	DLVP (mmHg)	MBP (mmHg)	HR (bpm)	Flow (mL/min)
Control (*X* ± SE)	1660.81 ± 214.11	−1193.98 ± 192.70	63.73 ± 3.86	12.63 ± 3.77	50.55 ± 0.14	241.38 ± 20	9.67 ± 0.40
DL-Hcy TLHC + DL-PAG (*X* ± SE)	1989.60 ± 230.29*	−1356.66 ± 219.02	62.65 ± 6.08	7.21 ± 2.64**	50.76 ± 0.14	205.46 ± 34.4	8.56 ± 0.34*

The values are expressed as the mean ± SE. *Statistical significance (*P* < 0.05), **high statistical significance (*P* < 0.01).

**Table 1d:** (d)

	*dp*/*dt* max (mmHg/s)	*dp*/*dt* min (mmHg/s)	SLVP (mmHg)	DLVP (mmHg)	MBP (mmHg)	HR (bpm)	Flow (mL/min)
Control (*X* ± SE)	1671.76 ± 94.89	−748.85 ± 67.71	57.30 ± 0.87	1.15 ± 0.45	49.70 ± 0.73	237.01 ± 8.13	10.50 ± 0.39
DL-Hcy TLHC + PPR IX (*X* ± SE)	819.91 ± 31.26*	−396.76 ± 29.62*	22.26 ± 2.48*	1.86 ± 0.44	50.05 ± 0.72	107.50 ± 7.20*	5.63 ± 0.38*

The values are expressed as the mean ± SE. *Statistical significance (*P* < 0.05).

**Table 2a:** (a)

	O_2_^−^ (nmol/mL)	H_2_O_2_ (nmol/mL)	NO_2_^−^ (nmol/mL)	TBARS (*µ*mol/mL)
Control (*X* ± SE)	19.97 ± 0.45	20.28 ± 0.04	23.14 ± 1.17	26.20 ± 0.38
DL-Hcy TLHC (*X* ± SE)	20.19 ± 0.47	20.23 ± 0.03	23.52 ± 1.13	25.88 ± 0.91

The values are expressed as the mean ± SE.

**Table 2b:** (b)

	O_2_^−^ (nmol/mL)	H_2_O_2_ (nmol/mL)	NO_2_^−^ (nmol/mL)	TBARS (*µ*mol/mL)
Control (*X* ± SE)	40.70 ± 6.24	34.55 ± 2.45	23.74 ± 1.00	27.02 ± 3.04
DL-Hcy TLHC + L-NAME (*X* ± SE)	23.33 ± 4.45*	24.70 ± 3.03*	23.30 ± 0.51	17.46 ± 2.63**

The values are expressed as the mean ± SE. *Statistical significance (*P* < 0.05), **high statistical significance (*P* < 0.01).

**Table 2c:** (c)

	O_2_^−^ (nmol/mL)	H_2_O_2_ (nmol/mL)	NO_2_^−^ (nmol/mL)	TBARS (*µ*mol/mL)
Control (*X* ± SE)	21.53 ± 3.74	30.78 ± 1.52	23.88 ± 1.61	22.50 ± 3.00
DL-Hcy TLHC + DL-PAG (*X* ± SE)	21.55 ± 4.18	29.89 ± 1.05	23.75 ± 1.40	11.97 ± 7.42*

The values are expressed as the mean ± SE. *Statistical significance (*P* < 0.05).

**Table 2d:** (d)

	O_2_^−^ (nmol/mL)	H_2_O_2_ (nmol/mL)	NO_2_^−^ (nmol/mL)	TBARS (*µ*mol/mL)
Control (*X* ± SE)	30.69 ± 4.97	25.28 ± 1.12	23.62 ± 0.53	21.54 ± 0.46
DL-Hcy TLHC + PPR IX (*X* ± SE)	16.84 ± 2.58	11.42 ± 0.26*	22.31 ± 0.80	20.96 ± 0.23

The values are expressed as the mean ± SE. *Statistical significance (*P* < 0.05).
